# Development and validation of a Satisfaction Questionnaire for Performance Appraisal Evaluation (SQPAE): a measurement instrument

**DOI:** 10.1186/s41155-025-00369-8

**Published:** 2026-01-05

**Authors:** Rosa Isabel Rodrigues, Ana Junça Silva, Cláudia Lopes

**Affiliations:** 1 Instituto Superior de Gestão – Business & Economics School, Lisbon, Portugal; 2 CIGEST – Management Research Center, Lisbon, Portugal; 3 ISCTE – Instituto Universitário de Lisbon, Lisboa, Portugal; 4 BRU - Business Research Unit, Lisboa, Portugal

**Keywords:** Performance appraisal, Satisfaction with the performance appraisal, Instrument development and validation, Measurement instrument, Human resources management

## Abstract

**Background:**

Performance appraisal (PA) plays a crucial role in human resource management by guiding professional development and informing decisions on promotions, compensation, and training. Employee satisfaction with the PA process is essential, as it affects motivation, engagement, and productivity. to effectively assess satisfaction with PA remain scarce.

**Main body:**

The Satisfaction Questionnaire for Performance Appraisal Evaluation (SQPAE) was developed to assess three core dimensions of satisfaction with PA: (a) the procedures involved, (b) the perceived effectiveness of the process, and (c) the feedback received.

**Objective:**

To develop and validate a reliable instrument for assessing employee satisfaction with PA and to identify its key contributing factors.

**Methods:**

The research comprised four studies.Study 1 focused on item development and expert validation. Study 2 applied exploratory factor analysis (EFA) to identify the structure and psychometric properties of the instrument. Study 3 confirmed the structure through confirmatory factor analysis (CFA) in an independent sample. Study 4 examined how the SQPAE components and different appraisal sources influence employee satisfaction. Data were collected via surveys in private-sector organisations, and statistical analyses were conducted using SPSS and AMOS.

**Results:**

The SQPAE demonstrated high reliability and validity. Feedback emerged as the strongest predictor of satisfaction with appraisal outcomes. Additionally, evaluations conducted by supervisors and peers were positively associated with satisfaction, while evaluations by subordinates showed a negative association.

**Conclusion:**

The SQPAE is a valid and reliable tool for assessing employee satisfaction with PA and offers a robust alternative to existing instruments. It can support organisations in improving PA processes to enhance employee engagement and performance. Future studies should examine its applicability across different organisational settings, hierarchical levels, and sectors.

## Introduction

Given the current uncertainty and complexity of the job market, organizations are under pressure to enhance their competitiveness (Jain, 2019). To address this challenge effectively, it was crucial for organizations to hire top professionals and assess whether they aligned with organizational needs and performed their tasks efficiently. This is where performance appraisal (PA) emerges as a strategic tool for human resource management (Nguyen et al., 2020). PA not only ensured that tasks were executed in the desired manner but also facilitated individual performance improvement and provided insights into each employee's contribution towards organizational goals (Okolie et al., 2020). Additionally, PA aided decision-making regarding other crucial human resource management practices such as compensation, training, and career development. Consequently, the concept of PA has evolved beyond a purely evaluative approach and was now seen as an integration of human resource management with organizational policies, shaping the strategic direction of the company (He et al., 2020). In this context, performance and motivational efforts were viewed from an improvement standpoint, leading to the transformation of PA models that emphasized competencies and objectives (Rahman et al., 2020). According to Rodrigues et al. (2023), PA plays a crucial role in human resource management, regardless of the adopted management model. It helps measure employees' performance, identify areas for development, and provides valuable information for decision-making related to compensation, promotions, and professional development.

In the present study, we aimed to develop and validate an instrument to effectively evaluate the level of worker satisfaction with their PA. The primary objective was to construct a comprehensive assessment tool that not only measured satisfaction but also identified the key contributing factors.

To achieve this goal, the validation process of the SQPAE was structured into four sequential studies. Study 1 focused on the development of the initial pool of items and content validation through expert review. Study 2 aimed to explore the underlying factor structure and psychometric properties of the instrument using exploratory factor analysis (EFA). Study 3 sought to confirm the factorial structure identified in Study 2, using confirmatory factor analysis (CFA) in an independent sample. Finally, Study 4 examined the relative impact of each component of the SQPAE on employees’ satisfaction with appraisal outcomes, as well as the influence of different sources of evaluation.

These factors encompassed the procedures employed throughout the PA process, their effectiveness, and the feedback provided to employees. Questionnaires provide a systematic and structured approach to performance assessment in the workplace, offering benefits related to fairness, objectivity, quantifiability, and organizational effectiveness. Therefore, it is important to develop instruments that accurately measure the quality of work performed so that they align with the organization's goals and values. Additionally, the construction of this questionnaire contributes to introducing a product that effectively does not exist and is essential for assessing the quality and quantity of work done (Nguyen et al., 2020). Okolie et al. (2020) add that it is the skills of the workers that contribute the most to achieving organizational success.

The construction and validation of this measurement instrument were carried out through a meticulous process involving three distinct studies. Additionally, a fourth study was conducted to specifically examine and determine the influential factors that impacted employee satisfaction with their PA. This comprehensive approach ensured a robust and thorough understanding of the subject matter, offering valuable insights for future improvements in the realm of PA satisfaction.

## Literature review

### Performance appraisal process

PA is recognised as an essential practice in people management, as it enables the identification of strengths and areas for development that employees need to address to meet the organisation’s objectives (Madureira et al., 2021). It also provides key information to support employees’ integration into the organisation and to promote continuous performance improvement (Al-Jedaia & Mehrez, 2020). As a result, it is designed to meet the needs of both employees and the organisation and aims to: (a) align workers' performance with the organization's strategy; (b) convey what is most valued by the organization; (c) assess workers' performance; (d) stimulate the acquisition of new skills; and (e) increase workers' motivation levels (Stewart & Brown, 2020).

Therefore, the PA process should consider three key domains: (a) task performance, which focuses on attitudes that contribute to improving productivity; (b) contextual performance, which concerns behaviors that go beyond work obligations (e.g., proactivity, cooperation) and help achieve the organization's goals; and (c) counterproductive behaviors, which refer to attitudes and actions that harm well-being in the workplace (Ramos-Villagrasa et al., 2019).

Regardless of the specific strategy adopted to evaluate employees, it is essential to understand their capabilities to perform assigned tasks, as well as their motivation to do so. Only by addressing both dimensions can organisations gain a competitive advantage and differentiate themselves in the job market (Deprá et al., 2021).

### Satisfaction with the performance appraisal

Employee satisfaction with PA is a critical factor in the organisational context, as it is directly associated with individual performance (Diamantidis & Chatzoglou, 2019; Setiawati & Ariani, 2020). When employees perceive the appraisal process as fair and impartial, satisfaction increases, reinforcing performance and organisational commitment (Memon et al., 2020). Conversely, dissatisfaction with PA can lead to negative attitudes and perceptions, potentially resulting in decreased productivity and higher turnover intentions (Memon et al., 2019).

PA is therefore fundamental to unlocking individual potential. Satisfaction with PA is based on a reciprocal dynamic: when employees perceive the process as fair and mutually beneficial, they tend to respond with greater engagement and improved performance. Furthermore, higher satisfaction with PA outcomes is associated with increased motivation and reduced intention to leave the organisation (Al-Jedaia & Mehrez, 2020).

As a key component of human resource management, PA is a systematic and periodic procedure that enables the analysis of employee performance by comparing results with organisational goals and identifying areas for improvement. It also serves as a key tool for professional development, offering feedback on performance and guidance for growth (Diamantidis & Chatzoglou, 2019). Al-Jedaia and Mehrez (2020) further highlight that identifying strengths and weaknesses supports informed decisions regarding promotions, training needs, and career progression. When well designed and implemented, PA contributes to the improvement of both individual and organisational performance (He et al., 2020).

According to Rodrigues et al. (2023), several factors influence employee satisfaction with PA, notably its perceived usefulness and accuracy, which are reflected in the effectiveness of the process and the quality of the feedback provided.

### Effectiveness of the performance appraisal process

The effectiveness of the PA process depends on several factors, including the clarity of evaluation objectives, the accuracy of the performance measures used, the objectivity of those conducting the appraisal, employees’ ability to interpret the feedback provided, and the usefulness of the results for performance improvement.

(Pichler, 2019). Organisations are therefore highly concerned with ensuring the effectiveness of their PA systems, particularly in the development of tools that optimize the process, conditions, and success factors (Igbal et al, 2019).

According to Cunha et al. (2018), PA involves aligning individual actions with organisational goals and values, making it essential for employees to understand their role and contribution to organisational success. If this alignment is not perceived, the system may become ineffective, generating resistance and internal conflict (Bayo-Moriones et al., 2020). When the PA system is effective, its integration within the organisation improves, and employees are more likely to view it as a meaningful contributor to their professional development (Beurden et al., 2020).

### Feedback of the performance appraisal process

PA provides structured and measurable information that supports employee growth and development. Feedback is considered a key component of PA, as it contributes to improving professional performance (DeNisi & Gonzalez, 2017). According to Rodrigues (2017), the characteristics of the feedback and the responses it elicits are among the most important elements of the PA process, as they offer clear guidance on areas that require improvement.

Feedback is considered a key component of PA, as it contributes to improving professional performance (DeNisi & Gonzalez, 2017). According to Rodrigues (2017), the characteristics of the feedback and the responses it elicits are among the most important elements of the PA process, as they offer clear guidance on areas that require improvement.

When feedback is provided constructively, employees feel motivated to grow and continuously improve their performance, resulting in increased motivation and productivity (Tagliabue et al., 2020). Gnepp et al. (2020) add that the uniqueness of each organization, task, and individual requires personalized feedback that can align both organizational and individual goals.

## Study 1—Development of the items of Satisfaction Questionnaire for Performance Appraisal Evaluation (SQPAE)

The items comprising the SQPAE were adapted from previously published scales in the literature, covering: (a) PA (Stathakopoulos, 1997; *e.g*., *I am satisfied with PA criteria*); (b) effectiveness of the PA process (Szlávic et al.,^2014^); *e.g*., *I am satisfied with the tasks included in my PA form*); (c) quality of feedback (Steelman et al., 2004; *e.g., I am satisfied that the feedback from my appraisal impacts my performance*); (d) reaction to feedback (Tuytens & Devos, 2012; *e.g*., *I am satisfied with the fact that the PA results lead to training activities*); (e) usefulness of PA (Greller, 1978; *e.g*., *I am satisfied with being promoted due to my excellent performance*); (f) accuracy of the PA (Stone et al., 1984; *e.g*., *I am satisfied with the way objectives are measured*), and (g) overall satisfaction with the PA process (Rodrigues et al., 2023; *e.g*., *I am satisfied with the results of my performance appraisal*). Despite the contributions of the aforementioned studies, none of them offers an instrument capable of simultaneously assessing all these dimensions, which highlights the relevance of developing the SQPAE.

**Fig. 1:**
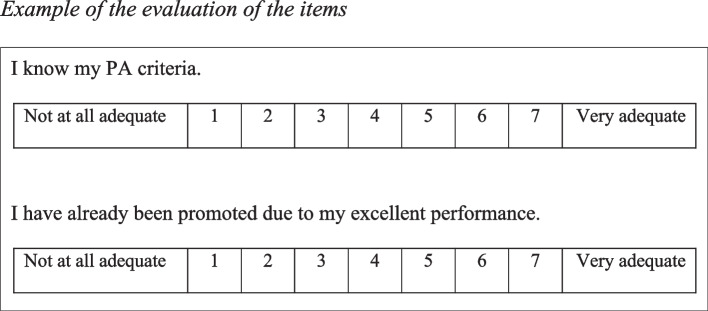
Example of the evaluation of the items

Based on the literature reviewed, 38 items were initially developed. These were then evaluated through a face-to-face feedback session involving experienced HR directors with over 15 years of professional experience. The items were rated on a seven-point Likert scale ranging from 1 (Not at all adequate) to 7 (Very adequate), with the aim of assessing their applicability to PA in a workplace context. Scores were calculated by summing the values assigned to each item (Fig. 1).

After collecting responses, only 20 items were deemed adequate. This selection followed the recommendation by Howell (2012), who suggests retaining items for which at least 75% of the responses fall within the top categories.

## Study 2—Exploratory study

The accuracy of the results depends considerably on the measurement instruments used, so it is crucial to assess their psychometric properties. Accordingly, we conducted a varimax-rotated Principal Component Analysis (PCA) to examine the correlations between variables and group them into components based on their similarities (Hongyu, 2018).

### Method

This is a cross-sectional study in which we collected the data through a questionnaire survey from a convenience sample. This sampling method was chosen due to the ease of access to participants (Mweshi & Sakyi, 2020).

### Sample

A total of 337 employees from various private-sector organisations participated in the exploratory study. The average age was approximately 38 years (Min = 19; Max = 69; SD = 11.29), and 50.1% of participants were women. More than half held a university degree or higher and worked in organisations with 250 or more employees (66.2%). The classification of companies followed Recommendation 2003/361/EC of the European Commission of 6 May 2003 (European Commission, 2003).^1^

### Procedures

The items assessing satisfaction with the PA process and the sociodemographic questions were entered into Google Forms. The link was distributed by email to the researchers’ professional contacts. The study was conducted in Portugal and received ethical approval from the Ethics Committee of ISG/CIGEST (Reference: CIG_0010/2025; Date: 5 March 2025). All procedures complied with the ethical standards of institutional and national research committees, as well as with the 1964 Helsinki Declaration and its later amendments. Participants were assured of full confidentiality and anonymity. Subsequently, the data were analyzed using the statistical software SPSS (Statistical Package for the Social Sciences, version 29.0).

## Results

### Construct validity

The PCA demonstrated the existence of a three-factor structure that explains 62.7% of the total variance (Table 1). The extraction of the components was based on three assumptions: (a) the Kaiser-Guttmann criterion; (b) the analysis of the scree plot; and (c) the percentage of variance explained (Furr, 2021). To further verify the number of factors to retain, we conducted a parallel analysis using 1000 random datasets and a 95th percentile threshold (O’Connor, 2000). The results showed that only the first two eigenvalues from the actual data (8.21 and 1.45) exceeded the corresponding simulated values (1.54 and 1.43, respectively), while the third eigenvalue (1.00) did not exceed the simulated threshold (1.36).

**Table 1:** Factorial matrix of the SQPAE after varimax rotation

I am satisfied with …	C 1	C2	C3
1. The access to the evaluation manual	.853		
2. PA criteria	.816		
3. Tasks included in my PA form	.761		
4. How the PA process is developed	.626		
5. Knowledge/training the people assessing me have in that area	.521		
6. The way objectives are measured	.487		
7. Freedom to express/defend my point of view during the appraisal interview		.793	
8. I can discuss the PA's objectives with my manager		.710	
9. The date of the feedback interview is scheduled in advance		.673	
10. My performance is monitored throughout the year		.670	
11. My objectives are realistic/achievable		.555	
12. Improvements shown in the PA when compared to previous evaluations		.517	
13. Feedback from my appraisal has an impact on my performance			.815
14. Fact that my salary depends on my performance			.779
15. Being promoted due to my excellent performance			.542
16. The fact that the PA results lead to training activities			.487
*Eigenvalue*	8.21	1.45	1.00
% variance explained	23.10	22.04	17.57
Cronbach Alpha	.86	.87	.76

*C1* Procedures of the PA process, *C2* Effectiveness of the PA process, *C3* Feedback of the PA process

Although these traditional criteria are widely used in exploratory factor analysis, we acknowledge that parallel analysis is considered a more robust technique for factor retention (Horn, 1965; Patil et al, 2008).

In this study, we opted for the traditional criteria due to their alignment with prior literature and the interpretability of the solution. Moreover, the three-factor structure was later supported by confirmatory factor analysis in Study 3. This decision was also grounded in the conceptual coherence of the three extracted dimensions – procedures, effectiveness, and feedback –, which are well established in the performance appraisal literature. Therefore, both theoretical interpretability and empirical confirmation justified retaining the three-factor model.”

We recommend that future validations of the SQPAE include parallel analysis to further substantiate the factor structure.

The Kaiser–Meyer–Olkin indicator (KMO =.93) and Bartlett's test of sphericity [χ2(253) = 3215.2, *p* <.001] also confirmed the adequacy of the factor analysis. It should be noted that four items were excluded: three due to high cross-loadings across all components and one due to a low item-factor correlation (below.400).

#### Reliability

Cronbach's Alpha coefficient was used to assess reliability. All components showed adequate internal consistency:.86 for the Procedures of the PA process;.87 for the Effectiveness of the PA process; and.76 for the Feedback of the PA process. Furthermore, it is important to highlight that the overall measurement, consisting of the 16 elements, demonstrated a coefficient of.93.

#### Normality Test

The mean value of satisfaction with PA was calculated by summing the 16 items. Component scores were computed by averaging the scores of their respective items. The Kolmogorov–Smirnov test (*n* = 337) showed that the distribution does not follow normality patterns (*p* <.001). Nevertheless, the central limit theorem revealed that the skewness and kurtosis coefficients are close to zero (Table 2), as they are within the reference values recommended by Demir (2022), which range between −1.96 and 1.96.

**Table 2:** Descriptive statistics

Global scale and components	*M*	*SD*	*CS*	*CK*
PA satisfaction (global scale)	4.33	1.25	- 1.31	-.98
Procedures of the PA process	4.51	1.44	- 1.90	-.48
Effectiveness of the PA process	4.48	1.32	- 1.81	- 1.21
Feedback of the PA process	3.79	1.49	0.37	1.74

*M* Mean, *SD* Standard-deviation, *CS* Coefficient of skewness, *CK* Coefficient of kurtosis

## Study 3—Confirmatory study

Study 3 aimed to test and confirm the factor structure of the SQPAE identified in Study 2, using confirmatory factor analysis (CFA).

### Sample

A total of 470 employees participated in this study, the majority of whom were female (56.4%). Their ages ranged between 18 and 68 years old (*M* = 38.4; *SD* = 11.6). Regarding their academic level, 26.8% of the participants had a degree, and 40.2% had a postgraduate qualification; 62.6% work for large companies.

### Procedures

Study 3 followed the same data collection procedures as Study 2. Before conducting the CFA, a preliminary study was conducted to test for multicollinearity. The results showed that the Tolerance values were higher than.20 and the VIF values were lower than 5, indicating the absence of multicollinearity and, consequently, of redundant items (Hair et al., 2019). Subsequently, the model’s fit was assessed using the Chi-square (χ^2^), Goodness of Fit Index (GFI), Comparative Fit Index (CFI), Standardized Root Mean Square Residual (SRMR), and Root Mean Square Error of Approximation (RMSEA).

### Results

To evaluate the questionnaire’s structure, two conceptual models were tested. Model 1: a single-factor model including all items; Model 2: a three-factor model based on the PCA results from Study 2. We followed the cut-off criteria suggested in the literature (e.g., Hair et al., 2019; Xia & Yang, 2018), which indicated that the three-factor model, with covariations suggested by AMOS modification indices, provided the best fit. Based on the model fit indices presented in Table 3, the three-factor model demonstrated the best fit to the data. We therefore retained this solution for subsequent analyses, given its statistical adequacy and conceptual interpretability [*χ*^2^_(109)_ = 3.76, *p* <.001, CFI =.91, GFI =.90, RMSR =.05, RMSEA =.07, LO90 =.06, HI90 =.08].

**Table 3:** SQPAE adjustment measures

	*χ*^2^/df	CFI	GFI	RMSR	RMSEA
1 factor	5.48	.81	.80	.07	.11
3 factors	6.03	.83	.84	.07	.10
Error covariance suggested by the AMOS modification indices					
	*χ*^2^/df	CFI	GFI	RMSR	RMSEA
1 factor	3.77	.89	.87	.05	.08
3 factors	3.76	.91	.90	.05	.07

Reliability was assessed using Cronbach’s alpha, which indicated high internal consistency for all three component: Procedures of the PA process (α =.84), Effectiveness of the PA process (α =.83), and Feedback of the PA process (α =.74). In addition, Composite Reliability (CR) exceeded.70 for all constructs, indicating good internal consistency (Table 4). The Average Variance Extracted (AVE) was above.50, supporting convergent validity. Discriminant validity was also confirmed, as both the Average Shared Variance (ASV) and the Maximum Shared Variance (MSV) were lower than the corresponding AVE values (Valentini & Damásio, 2016).

**Table 4:** Composite reliability, convergent and discriminant validity

Variables	CR	AVE	MSV	ASV
Procedures of the PA process	.83	.68	.61	.41
Effectiveness of the PA process	.71	.64	.57	.44
Feedback of the PA process	.81	.72	.62	.42

*CR* Composite reliability, *AVE* Average variance extracted, *MSV* Maximum shared variance, *ASV* Average shared variance

Taken together, the findings across the different studies indicate that the SQPAE exhibits strong psychometric properties, supporting its use as a valid and reliable alternative to existing instruments in PA processes.

## Study 4—Factors that influence satisfaction with the performance appraisal process

Given the robust psychometric properties of the SQPAE, Study 4 aimed to examine which of its components had the strongest impact on employees’ satisfaction with PA outcomes, and how different sources of evaluation influenced this satisfaction. This enabled us to explore the instrument’s predictive capacity and practical relevance in organisational contexts.

Employee satisfaction with PA may also depend on the source of the evaluation. Prior studies have shown that self-assessment, supervisor feedback, and peer reviews can influence how employees perceive the fairness and usefulness of the appraisal process (Mercer, 2019; Murphy, 2019). In this study, we analysed the impact of different evaluator sources on satisfaction with PA results.

Evaluation by direct supervisors is often seen as a key source of feedback, as supervisors are best positioned to observe and assess employee performance. However, limited availability for continuous observation may reduce the perceived fairness of the PA process, ultimately lowering satisfaction (Brenan, 2020).

Peer evaluation may provide valuable insights, given colleagues’ familiarity with day-to-day performance. Yet peers may lack the authority or training to issue formal judgments. Moreover, competition for promotions and rewards can introduce bias (Vozza, 2020).

Subordinate evaluation is used less frequently, partly due to concerns about undermining managerial authority or fear of retaliation, which may distort results (Villeval, 2020). Some organisations also involve other stakeholders (e.g., customers, suppliers) in the appraisal process—particularly in customer-facing roles—resulting in a 360-degree feedback model (Campion et al., 2019). Satisfaction with PA can significantly impact employee motivation, commitment, and performance, which underscores the need for fair and transparent systems that incorporate diverse sources of evaluation (Aguinis & Burgi-Tian, 2021).

### Sample

A total of 450 professionals participated, 54.7% of whom were female. Ages ranged from 19 to 67 years (M = 38.8; SD = 11.2). Of the participants, 25.3% held a university degree, and 41.3% had postgraduate qualifications in the social sciences (e.g., psychology, economics, sociology). Additionally, 71.3% worked in large companies, with 69.8% in the services sector.

Regarding the PA process, 49.8% of the participants reported being evaluated annually, 26.7% every two years, and 23.6% semi-annually. Only 18.9% of the participants engaged in self-assessment, and none reported being evaluated using a 360-degree feedback model.

### Procedures

Study 4 followed the same data collection procedures as Study 2. The questionnaire was distributed via Google Forms through the researchers’ professional networks. Participants were informed about the study’s objectives, and confidentiality and anonymity were guaranteed. In addition to completing the SQPAE, participants were asked to indicate which source(s) conducted their PA (e.g., supervisor, peers, self-assessment, subordinates). Data analysis was performed using SPSS version 29.0.

### Results

Statistically significant differences were found in SQPAE components based on PA frequency: procedures [*F*_(2, 447)_ = 2.429, *p* < 0.05], effectiveness [*F*_(2, 447)_ = 4.992, *p* < 0.05], and feedback [*F*_(2, 447)_ = 16.619, *p* < 0.001].

Employees evaluated semi-annually reported higher satisfaction levels across all SQPAE dimensions compared to those evaluated annually or biennially. Satisfaction was measured using a seven-point Likert scale (1 = very dissatisfied to 7 = very satisfied), with composite scores obtained by summing the items in each dimension. Higher scores reflect greater satisfaction.

To identify the SQPAE component with the strongest influence on satisfaction with PA outcomes, we used a single-item measure [*I am satisfied with the results of my PA*; Rodrigues et al., 2023). While single-item measures are sometimes criticised for lacking psychometric robustness, they are widely accepted in organisational contexts where brevity and interpretability are important (Sedlak, 2020). Fakunmoju (2020) also highlights their practical advantages over longer multi-item scales.

**Fig. 2:**
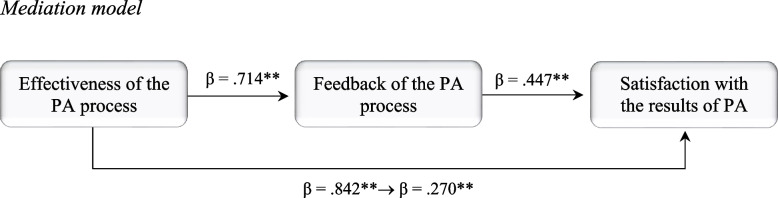
Mediation model

The three SQPAE components together explained 44.3% of the variance in satisfaction with PA results. Only feedback (β =.427, *p* <.001) and effectiveness (β =.253, *p* <.001) significantly predicted satisfaction, while procedures showed no significant effect. Based on these results, we tested whether feedback mediated the relationship between perceived effectiveness and satisfaction with PA outcomes (Fig. 2).

The mediation analysis revealed that introducing feedback as a mediating variable reduced the effect of effectiveness on satisfaction, although the relationship remained significant. The regression coefficient decreased from *β* =.842, *p* < 0.001 to *β* =.270 (*p* < 0.001) indicating partial mediation. This indirect effect was statistically significant (Sobel Z = 8.214, *p* < 0.001; Preacher, 2023). The positive coefficient suggests that greater perceived feedback quality enhances the relationship between effectiveness and satisfaction.

Finally, we examined which evaluation sources were most predictive of satisfaction. As shown in Table 5, only supervisor evaluation (*β* =.117, *p* < 0.05) and peer evaluation (*β* =.199, *p* < 0.001) had a positive and significant influence on satisfaction with PA outcomes.

**Table 5:** Influence of evaluation sources on satisfaction with the results of performance appraisal

Predictive variables	Satisfaction with the results of PA (β)
Self-Assessment	.005
Supervisor	.117*
Peers	.199**
Subordinates	-.311
	Adjusted R^2^.035
	*F*_(4, 445)_ 5.097**

These findings reinforce the importance of multi-source feedback in enhancing satisfaction with PA, particularly when it originates from supervisors and peers.

## Conclusions

Employees’ satisfaction with PA is essential for maximising organisational talent (Memon et al., 2019). However, it is found that satisfaction levels can be influenced by various factors, including the procedures used during the PA process, its effectiveness, and the feedback received from it (Rodrigues et al., 2023). Developing valid and accurate tools to assess satisfaction with PA is therefore critical, as this satisfaction is closely linked to job performance (Memon et al., 2020).

The development of such an instrument requires a continuous process of conceptual and technical refinement, involving a set of procedures to ensure that the items accurately reflect the construct being measured (Fernández-del-Río et al., 2019). Accordingly, three studies were conducted to: (a) identify the questionnaire structure (number and nature of factors specified through EFA and/or CFA); (b) study internal consistency (e.g., Cronbach's alpha coefficient, item correlations); (c) analyze the content homogeneity of each dimension; (iv) include items that discriminate subjects' responses; and (v) confirm the psychometric properties of the instrument (Taherdoost, 2019).

The internal consistency indices for the three extracted dimensions exceeded.70 in both Study 2 and Study 3, indicating good reliability. The obtained KMO value reflected adequate sampling adequacy (Marôco, 2021), and the CFA showed no evidence of multicollinearity or redundant items, confirming a good model fit (Xia & Yang, 2018).

Study 4 extended the validation of the SQPAE, showing that specific components of the instrument, particularly feedback and perceived effectiveness, significantly influence employees’ satisfaction with appraisal outcomes. Furthermore, the study highlighted the role of different evaluation sources in shaping satisfaction levels.

Although the instrument demonstrated good internal consistency and structural validity, one limitation is that concurrent and predictive validity were not assessed. Future research should address this gap by comparing SQPAE scores with external performance indicators or longitudinal outcomes, in order to strengthen the instrument’s robustness. In addition, the absence of data on participants’ most recent performance appraisal ratings limited our ability to cross-check perceptions with objective indicators.

One limitation of this study is that no data were collected regarding participants’ most recent PA ratings. As a self-administered questionnaire, the instrument captures employees’ perceptions rather than their actual performance. Moreover, this was a cross-sectional and self-reported study, which may introduce some bias due to the absence of control for social desirability.

To mitigate common method bias, we adopted the procedures recommended by Jordan and Troth (2020), including: (a) clarifying the research objective and providing clear instructions to participants; (b) ensuring that the questions were formulated in a concise, simple, and unambiguous manner; and (c) ensuring the use of a precise and easily interpretable scale.

We also conducted Harman’s single-factor test, which yielded a solution with one factor explaining 25.8% of the total variance (eigenvalue > 1 = 4.8). This suggests that common method variance did not substantially influence the results.

It should also be noted that the sample was based on convenience sampling, which limits the possibility of generalising the findings to the broader population. Moreover, participants were exclusively employees from large private-sector organisations. This may affect generalisability, as perceptions of PA satisfaction could differ in public institutions or smaller companies. Future studies should replicate the validation process in varied organisational contexts.

The SQPAE can be integrated into performance management systems to regularly assess employee perceptions and satisfaction with the appraisal process. Its results can support decisions related to feedback practices, the redesign of appraisal procedures, and the alignment of evaluation tools with employee expectations.

Although this instrument was validated within a Portuguese context, its conceptual foundations are grounded in international literature. We therefore recommend that future studies explore its cross-cultural applicability, including translation, cultural adaptation, and psychometric validation in diverse organisational and national settings.

In summary, the SQPAE not only fills a critical gap in the measurement of PA satisfaction but also offers a valuable diagnostic tool for enhancing human resource practices in contemporary organisations.

## Data Availability

The data underlying this article will be shared on reasonable request to the corresponding author.

## Abbreviations

ASV: Average shared variance
AVE: Average Variance Extracted
CFA: Confirmatory Factor Analysis
CFI: Comparative Fit Index
CK: Coefficient of kurtosis
CR: Composite Reliability
CS: Coefficient of skewness
EFA: Exploratory Factor Analysis
GFI: Goodness of Fit Index
KMO: Kaiser–Meyer–Olkin indicator
M: Mean
MSV: Maximum shared variance
PA: Performance Appraisal
PCA: Principal Component Analysis
RMSEA: Root Mean Square Error of Approximation
SD: Standard-deviation
SQPAE: Satisfaction Questionnaire for Performance Appraisal Evaluation
SRMR: Standardized Root Mean Square Residual
χ^2^: Chi-square

## Footnotes

1 Small company = Less than 49 employees; Medium company = Between 50 and 249 employees; Large company = More than 250 employees.
